# Vulnerabilities of Local Healthcare Providers in Complex Emergencies: Findings from the Manipur Micro-level Insurgency Database 2008-2009

**DOI:** 10.1371/currents.dis.397bcdc6602b84f9677fe49ee283def7

**Published:** 2013-04-24

**Authors:** Samrat Sinha, Siddarth David, Martin Gerdin, Nobhojit Roy

**Affiliations:** Centre for Study of Political Violence, Jindal School of International Affairs, O.P.Jindal Global University, Sonipat, Haryana, India; Centre for Enquiry into Health and Allied Themes (CEHAT), Mumbai, India; Health Systems and Policy, Department of Public Health Sciences, Karolinska Institutet, Stockholm, Sweden; Jamsetji Tata Centre for Disaster Management, Tata Institute of Social Sciences, Mumbai, India

## Abstract

Background: Research on healthcare delivery in zones of conflict requires sustained and systematic attention. In the context of the South Asian region, there has been an absence of research on the vulnerabilities of health care workers and institutions in areas affected by armed conflict. The paper presents a case study of the varied nature of security challenges faced by local healthcare providers in the state of Manipur in the North-eastern region of India, located in the Indo-Myanmar frontier region which has been experiencing armed violence and civil strife since the late 1960s. . The aim of this study was to assess longitudinal and spatial trends in incidents involving health care workers in Manipur during the period 2008 to 2009. Methods: We conducted a retrospective database analysis of the Manipur Micro-level Insurgency Database 2008-2009, created by using local newspaper archives to measure the overall burden of violence experienced in the state over a two year period. Publicly available press releases of armed groups and local hospitals in the state were used to supplement the quantitative data. Simple linear regression was used to assess longitudinal trends. Data was visualized with GIS-software for spatial analysis. Results: The mean proportion of incidents involving health care workers per month was 2.7% and ranged between 0 and 6.1% (table 2). There was a significant (P=0.037) month-to-month variation in the proportion of incidents involving health care workers, as well as a upward trend of about 0.11% per month. Spatial analysis revealed different patterns depending on whether absolute, population-adjusted, or incident-adjusted frequencies served as the basis of the analysis. Conclusions: The paper shows a small but steady rise in violence against health workers and health institutions impeding health services in Manipur’s pervasive violence. More evidence-building backed by research along with institutional obligations and commitment is essential to protect the health-systems Keywords: India, Manipur, insurgency, healthcare, security, ethnic strife

## Background

One of the characteristic features of current conflicts is the increasing number of non-combatants being caught in the violence as well as the commonality of attacks on civilian targets. There is reporting across the world of health facilities and their staff being attacked in conflict zones, clinics being bombed or demolished, healthcare workers being abducted or murdered, threats and intimidation against fulfilling medical duties which jeopardize lives of health-workers, disrupt services and affect access to medical care.^1^^,^ 2 Despite being part of the setting of armed conflict for decades such violence against healthcare providers and facilities is grossly overlooked,^3^^,^ 4 compounded by limited reporting, lack of impact-analysis and absence of mechanisms to prevent them.^3^ Events which even do receive some attention mainly involve international humanitarian workers featuring prominently in major news sources^5^ and have becoming subject to a landmark study.^6^

In contrast, less focus is directed towards security conditions that threaten the average working day of local healthcare workers, especially in a conflict that is not recognized internationally. Moreover on a country-level, non-state armed groups, out of the ambit of international protocols flout restrictions on protection to medical systems and workers due to asymmetrical nature of insurgency and counter-insurgency warfare.^7^ While country based studies of such threats do exist especially in cases such as Colombia,^8^ Iraq,^9^ Palestine,^10^ Mexico,^11^ and Nepal^12^ there is a dire need for enhanced data on the security risks for national health providers, especially local health providers at the sub-district level because they are the ones providing the everyday healthcare to conflict affected populations and the conflict situation leads to adverse effects on the their functioning.^13^

While there are protocols and guidelines^14^ for humanitarian organizations evolved at the organizational-level in conflict areas for staff protection, there is little recognition and consequently little work done on the protection of local health services and providers at the hospital-level. Therefore, documentation and research on the vulnerabilities of health care workers and facilities in conflict areas can help overcome this blind-spot. This is crucial from a public health perspective to enable planning at the policy-level, in order to protect local health facilities and providers. This paper attempts bridge this lacunae in evidence for addressing the risks on healthcare in armed conflict thereby helping in risk-estimation, recognizing trends, planning and action.

The North-eastern Indian state of Manipur which is situated in the Indo-Myanmar frontier region has been experiencing protracted conflict and insecurity since it was forcefully “merged” with the Indian Union on 15th October 1949. There were three underground organizations then, and today the number of Non-State Armed Groups (NSAG) has risen to 37 factions or more.^15^ Even though the situation in Manipur is not yet recognized as an armed conflict by the government,^16^ in 1980, the *Armed Forces Special Powers Act* (AFSPA) was introduced in the State which allows the security forces to undertake counterinsurgency operations with impunity.^17^

The events of the conflict are rarely reported in the mainstream national media, and there have been no precise estimates on the number of fatalities and injuries occurring as a result of the conflict. According to the Institute of Conflict Management (based on figures provided by the Ministry of Home Affairs) 5776 persons have been killed in the conflict between 1992 and 2012 with no data available on injuries.^18^ Blunted by the chronic disruptions to the healthcare delivery mechanism in the state, which has witnessed protracted intra-state conflict, the challenges to healthcare providers are under the radar and invisible. This is thus the first attempt to elaborate and systematically describe the nature of insecurity experienced by local health providers in the state. It seeks to complement studies on the safety and security of humanitarian workers by systematically analyzing the threats to national (and provincial) health systems in conflict zones drawing attention to this really significant gap in knowledge when it comes to local health systems in conflict zones. The aim of this study was to assess longitudinal and spatial trends in incidents involving health care workers in Manipur during the period 2008 to 2009.

## Methods

Study design

This study uses a micro-level approach to record attacks on national health workers. The micro-level event-centred approach refers to the “decomposition of a conflict into discrete political and violent events, examination of the mechanisms through which they affect behaviour, and consideration of differential risks within the population”.^19^ It is a retrospective database analysis, part of the larger research project Manipur Micro-level Insurgency Database (MMID 2008-2009) involving longitudinal trend analysis. The MMID is a researchable database of 2538 insurgency related events and describe on a daily basis, incidents that are directly connected to the insurgency that occurred between September 2007 and December 2009. Incidents recorded in the database include lethal and non-lethal events. These non-lethal incidents include events such as the closures of schools, hospitals, attacks on government institutions, arrests, and abductions. A variety of sources were used to triangulate and ensure the verifiability of the information being recorded.

Setting

The state of Manipur is divided into nine districts: four “valley” districts Imphal East, Imphal West, Thoubal and Bishnupur and five “hill” districts , Chandel, Churachandpur, Senapati, Tamenglong, and Ukhrul, with a population of 2.7 million.^20^ Manipur has been populated by diverse hunger, governance and livelihoods.^21^ In the late seventies, many educated youth joined the ranks of nascent revolutionary linguistic and religious groups, including a number of indigenous tribes and ethnic groups. Especially important from the perspective of the conflict are the Metieis, Nagas and Kukis. Their survival is not only threatened by violence but also by multiple overlapping issues concerning health, organizations and engaged in an armed insurrection for an “Independent Manipur.”

Prominent among the NSAGs are the groups functioning in the “valley” and “hill” districts. The important “valley” based and mainly Metei dominated armed groups include the UNLF (United National Liberation Front), KYKL (Kanglei Yawol Kanna Lup), PREPAK (People’s Revolutionary Party of Kangleipak), KCP (Kangleipak Communist Party), PLA (Peoples Liberation Army) and PULF (Peoples United Liberation Front). Among the “hill” based groups the major are the Naga dominated NSCN-IM (National Socialist Council of Nagaland-Isaac-Muviah), NSCN-K (National Socialist Council of Nagaland-Khaplang) and 18 groups that make up the Kuki National Organization.

It must be pointed out that the situation is not only marked by armed insurrection. Driving the conflict dynamics are deep seated structural issues that are closely linked to ethnicity, development entitlements and the incompatibilities arising due to the imposition of modern land-tenure systems on traditional tribal arrangements. Indeed, it has been shown that there exists a degree of imbalance in the distribution of development entitlements between the “hill” and “valley” districts, whereby the more inaccessible hill districts, in which several of the minority tribes live, are deprived in terms of the non-implementation of various government development programmes.^22^

The nature of the state response and the type of security regime that governs the state is also problematic. The Government of India has labelled the Manipur conflict as a “law and order” problem” and the state is currently categorised as a “disturbed” area where there are ongoing military operations.^17^ There are several legal regimes beyond the prominent *Armed Forces Special Powers Act* that allow for the operations of the security forces with impunity.^23^ Human rights abuses by state and non-state actors have been well documented and include: enforced disappearances, arbitrary detention, sexual violence, extrajudicial killings and torture.^24^

Variables

For the longitudinal analysis, our primary outcome variable or dependent variable was the proportion of incidents involving health care workers per month. We defined an incident involving health care workers as any insurgency-related incident reported in MMID as affecting health care workers or facilities. The independent variable was time from start of the study period, counted in months. We included the monsoon season as a potential confounder and defined this season as starting June 1 and ending September 30. For the spatial analysis, the numbers of incidents involving health care workers were analyzed per district in Manipur. We calculated population-adjusted frequencies (per 100,000 population), incident-adjusted frequencies (per 100 incidents), and absolute frequencies and visualized the adjusted and absolute frequencies using Quantum GIS software (Quantum GIS 1.73, http://www.qgis.org/).

Data sources

The MMID relies primarily on locally published sources that report insurgency related events. Studies done in other low-income country conditions have found that local media sources contain rich contextual information and as they are closer to the events that they report.^25^ A hallmark of the database is that a majority of the events are not reported in national sources because they occur in remote areas and at the village level. The main sources of the database were archives of a state-level newspaper *TheImphal Press* as well as the records of a local online forum *E-Pao *that publishes stories from *The Sangai Express* and *Hueyien Lanpao* (which are both local newspapers). Population data for standardization of incidents per district was retrieved from the 2011 Indian census.^20^

Statistical methods

We used a simple linear regression model to assess longitudinal trends in incidents involving health care workers in Manipur during the period 2008 to 2009, the methodology mainly adopted from segmented linear regression.^26^^,^ 27 The dependent variable was calculated by dividing the number of incidents involving health care workers with the total number of incidents for each month. We excluded September 2007 from the analysis because of its small number of data points. The monsoon season was accounted for by including a dummy variable in the regression model.

We interpreted a time-co-effecient with *P*-value<0.05 as indicative of significant month-to-month variation in proportion of incidents involving health care workers. We defined a significant trend-deviation as a proportion of incidents involving health care workers falling outside the 95% confidence interval (CI) of the predicted proportion for that month. For visual interpretation, we superimposed a connected scatter plot of proportions per month on a range area plot of lower and upper confidence limits. Number of incidents with proportion estimates for each month is presented in table 1. We used the Durbin-Watson test to test for first-order auto-correlation; however no auto-correlation was found in our data. The Stata statistical software (Stata 12.0, StataCorp, Texas) was used for all statistical analysis. We applied a significance level of 5% and a confidence level of 95% to all statistical tests.

## Results

In total, 54 (2.1%) out of 2547 incidents recorded in the MMID 2008-2009 involved health care workers (table 1). The mean proportion of incidents involving health care workers per month was 2.7% and ranged between 0 and 6.1% (table 2). Linear regression analysis revealed a significant (*P*=0.037) month-to-month variation in the proportion of incidents involving health care workers, as well as a upward trend of about 0.11% per month (table 3). No month had a proportion of incidents involving outside the 95% CI of modelled proportions (figure 1).

**Table 1. Chronology of incidents involving healthcare workers in Manipur:** 

Date of incident	Type of incident	Location of incident
11^th^ December 2009	Bomb recovered from Secretary, Population Health Institution	Imphal
17^th^ January 2008	Shooting and Injury of Hospital Attendant	Imphal
18^th^ January 2008	Closure of Langol Hospital and Imphal Hospital (both private hosiptals) due to extortion	Imphal
5^th^ February 2008	Arrest of armed group member from RIMS Hospital Guardroom	Imphal
1^st^ March 2008	Grenade placed at residence of Director of RIMS Hospital	Imphal
14^th ^April 2008	Grenade recovered from residence of doctor	Imphal
25^th^ May 2008	Bomb Blast at RIMS hospital	Imphal
16^th^ June 2008	Arrest of armed group member in hospital premises, who was hospitalized	Imphal
3^rd^ July 2008	Bomb attack against residence of Chief Accountant, RIMS hospital	Imphal
4^th^ July 2008	Grenades recovered at residence of Superintendent, RIMS Hospital	Imphal
13^th^ July 2008	Bomb Attack against residence of doctor	Imphal
17^th^ July 2008	Bomb Attack against residence of lady doctor	Imphal
24^th^ July 2008	Grenade found at residence of doctor	Imphal
12^th^ September 2008	Temporary Closure of State run JN Hospital due to extortion demands	Imphal
18^th^ September 2008	Arrest of Manager and A Doctor of Raj Polyclinic	Imphal
19^th^ September 2008	Grenade recovered from Kangleipak Nursing Institute	Imphal-East
26^th^ September 2008	Grenade recovered from Kangleipak Nursing Institute	Imphal-East
28^th^ September 2008	Grenade attacks against Kangleipak Nursing Institute	Imphal-East
11^th^ October 2008	Firing on residence of RIMS doctor, serving in surgery ward	Imphal
12^th^ October 2008	Firing on residence of RIMS doctor, maid injured	Imphal
13^th^ October 2008	Person arrested for extortion from retired doctor	Imphal
25^th^ October 2008	Firing on residence of doctor	Imphal
29^th^ October 2008	Closure of Pharmacies around RIMS hospital in order to protest extortion threats	Imphal
1^st^ November 2008	Firing on residence of doctor	Imphal
7^th^ November 2008	Arrest of person for engaging in extortion from Babina Clinic and Imphal Hospital	Imphal
9^th^ December 2008	Abduction of two Engineers of RIMS Hospital	Imphal
11^th^ December 2008	Closure of Outpatient Department , Casualty Ward , and Operations Theatre of RIMS hospital due to above abductions	Imphal
16^th^ March 2009	Grenade recovered from Babina clinic	Imphal
18^th^ March 2009	Bomb blasts at Suba Hospital and Assisted Reproduction Centre, three injured	Imphal
16th April 2009	Closure of private hospital after ban by armed group	Imphal
21^st^ April 2009	Grenade recovered from private clinic, ACS Hospital	Imphal
28^th^ April 2009	Person arrested for engaging in extortion from Imphal Hospital	Imphal
2^nd^ May 2009	Closure of 40 medicine shops due to extortion	Imphal
13^th^ May 2009	Closure of medicine wholesalers and pharmacists due to extortion threats	Imphal
16^th^ May 2009	Bomb attack at RIMS Hospital	Imphal
9^th^ July 2009	Bomb blast at Raj Polyclinic, one attendant injured	Imphal
9^th^ July 2009	Bomb blast on road connecting Suba Hospital and RIMS hospital	Imphal
10^th^ July 2009	Bomb attack against residence of owner Raj Polyclinic, 2 injured	Imphal
18^th^ July 2009	Firing on RIMS hospital by unknown gunmen	Imphal
28^th^ July 2009	Firing and grenade attack on Langol View clinic, 2 civilians injured	Imphal
21^st^ August 2009	Bomb explosion at private hospital, 3 injured, 1 person arrested. The arrested person was serving in security forces	Imphal
27^th^ August 2009	Bomb recovered from residence of doctor, Chamber of Commerce Hospital	Imphal
1^st^ September 2009	Grenade recovered from residence of engineering consultant, RIMS Hospital	Imphal
29^th^ September 2009	Grenade attack against Langol View Clinic, private security guard injured	Imphal
10^th ^September 2009	Destruction of Public Health Centre (PHC) by armed group. The PHC was formerly occupied by the security forces	Thoubal District
4^th^ October 2009	Two Persons arrested for extortion against doctor	Imphal
21^st^ October 2009	Killing of former Chief Medical Officer by unknown gunmen	Imphal
28^th^ October 2009	Closure of all Hospitals to protest killing of former Chief Medical Officer and extortion demands	In Imphal and adjoining districts
31^st^ October 2009	Bomb blast at Imphal Hospital	Imphal
14^th^ November 2009	Grenade recovered from residence of Managing Director, Imphal Hospital	Imphal
7^th^ December 2009	Bomb recovered from Executive Engineer, Public Health Engineering Department (PHED)	Imphal
23^rd^ December 2009	Closure of routine surgeries at Shija Hospital due to disturbances on National Highway-39	Imphal
24^th^ December 2009	Arrested of woman armed group member for extortion against hospital	Imphal
27^th^ December 2009	Grenade attack against private hospital, five injured, including two security personnel	Imphal

**Scatter plot of the proportion of incidents involving health care workers.**
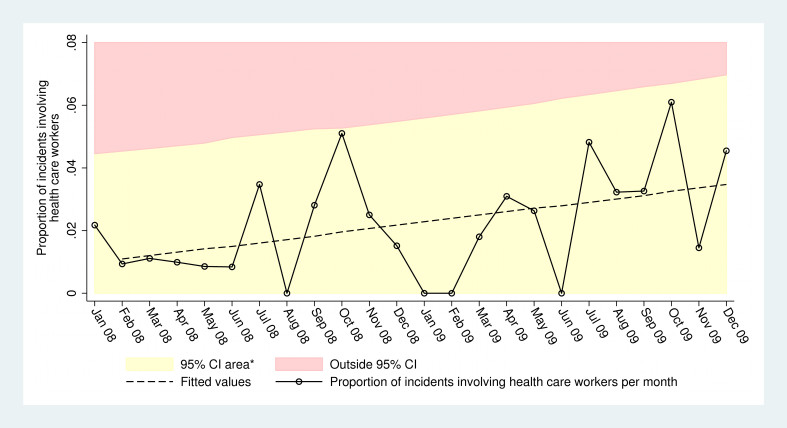
* 95% CI lower limit has been cut at 0 to allow for more intuitive interpretation of graph. Abbreviations: CI=Confidence Interval.

Spatial analysis revealed different patterns depending on whether absolute, population-adjusted, or incident-adjusted frequencies served as the basis of the analysis (figure 2). When frequencies were adjusted for population size, Chandel appeared to be most heavily burdened (figure 2A). In contrast, when frequencies were adjusted for total number of incidents peripheral districts appeared to be most heavily burdened (figure 2B). Finally, when absolute frequencies were considered our results indicated that districts around the capitol Imphal were most heavily burdened (figure 2C). District characteristics are reported in table 4.

**Table 2. Data characteristics and variables included in linear regression analysis:** *Variable included in linear regression analysis; **The proportion was included as a fraction of 1.

Actual month	Time from start of study period*	Monsoon*	Total number of incidents	Number of incidents involving health care workers	% of incidents involving health care workers*,**
January 2008	1	0	92	2	2.2
February 2008	2	0	107	1	0.9
Mars 2008	3	0	90	1	1.1
April 2008	4	0	101	1	1.0
May 2008	5	0	117	1	0.9
June 2008	6	1	119	1	0.8
July 2008	7	1	144	5	3.5
August 2008	8	1	137	0	0.0
September 2008	9	1	178	5	2.8
October 2008	10	0	98	5	5.1
November 2008	11	0	80	2	2.5
December 2008	12	0	132	2	1.5
January 2009	13	0	113	0	0.0
February 2009	14	0	98	0	0.0
Mars 2009	15	0	111	2	1.8
April 2009	16	0	97	3	3.1
May 2009	17	0	114	3	2.6
June 2009	18	1	103	0	0.0
July 2009	19	1	83	4	4.8
August 2009	20	1	62	2	3.2
September 2009	21	1	92	3	3.3
October 2009	22	0	82	5	6.1
November 2009	23	0	69	1	1.4
December 2009	24	0	110	5	4.5

**Table 3. Results of simple linear regression model:** *P<0.05. ^1^Included as potential confounder. Abbreviations: CI=Confidence Interval

**Independent variables**	**Coefficient**	**Standard error**	***P-*value**	**95% CI**
Constant	0.0077	0.0075	0.317	-0.0079 - 0.0233
Time from start of study period*	0.0011	0.0005	0.037	0.0001 - 0.0021
Monsoon^1^	-0.0003	0.0071	0.963	-0.01516 - 0.0145

**Table 4. District characteristics:** 

**District**	**Total population**	**Total number of incidents**	**Number of incidents involving health care workers**
Bishnupur	240363	92	1
Chandel	144028	147	5
Churachandpur	271274	46	1
Imphal East	452661	476	9
Imphal West	514683	385	10
Senapati	354972	23	0
Tamenglong	140143	26	1
Thoubal	420517	387	7
Ukhrul	183115	30	1

## Analysis

Acts of violence against healthcare are only the final outcomes of a longer chain of events that involve a degree of threats and negotiations. Moreover, the incidents extracted from the database are problematic, not because they seem to be few, but rather because they are deliberate and planned actions. In the case of RIMS hospital for instance, the institution which is the state-run nodal super-speciality for the entire region has been facing continuous interference in the construction of buildings, the appointment of staff and the admission of new students. Our results indicate that while the proportion of incidents involving health care workers varied significantly over time, no month deviated more than ±2 standard deviations from predicted values. This indicates that while it might be difficult to reliably predict the coming month’s toll to health care workers, over time the trend seems to be increasing.

**Manipur with frequencies of incidents involving health care workers mapped.**
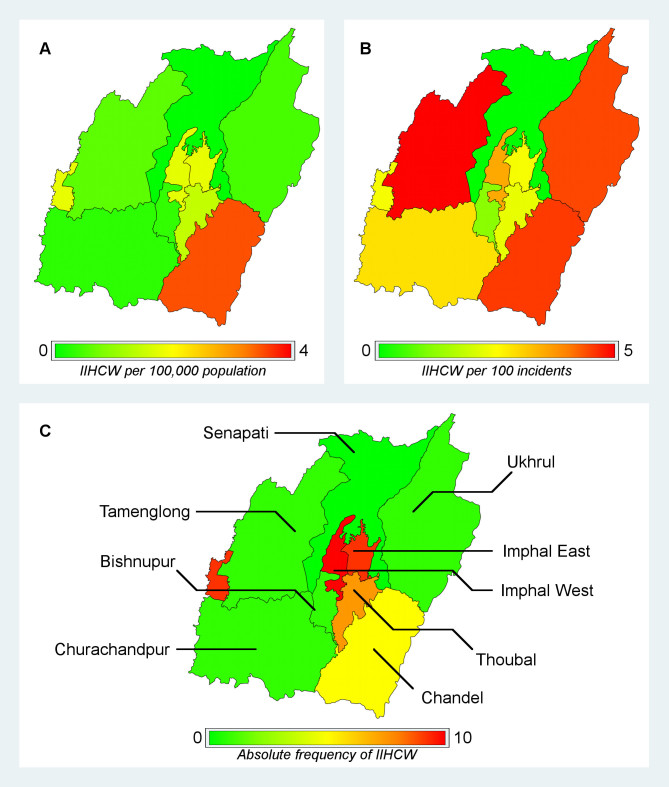
A) Population-adjusted frequencies of incidents involving health care workers. Frequencies are reported per 100,000 population. B) Incidence-adjusted frequecies of incidents involving health care workers. Frequencies are reported per 100 incidents.C) Absolute frequencies of incidents involving health care workers. Abbreviations: IIHCW=Incidents Involving Health Care Workers.

The spatial distribution of incidents above does not provide a complete picture. The issue must be looked at from two additional perspectives. While, the reasons for the concentration of attacks on healthcare in the capital is of concern, the manner by which health provision functions in the areas beyond the capital, especially the “hill” districts must also be taken into account. Also to be considered are the impacts of inter-ethnic tensions that obstruct healthcare through the various blockades. In other words, the issue of insecurity impinging on the functioning of the health systems actually has three components: first, in terms of threats to physical safety of healthcare providers and attacks on institutions which occurs in the capital region; secondly, the process by which weak governance, geographical remoteness and insecure conditions lead to the reluctance of health workers to function in areas that are remote and far away from the capital; and, third the manner by which healthcare provision becomes strained due to inter-ethnic tensions. While the first aspect of insecurity can be measured, the other two types of insecurity require a more systematic study and the evolution of distinct methodologies.

Three major categories of incidents occurred frequently: first, we see the closure of health institutions, including private clinics, medicine shops and hospitals; second, we see the use of grenades as intimidation tools whereby they are placed in residences and offices of health providers accompanied by warnings; and third, a high frequency of bomb attacks against health institutions or the residences and offices of health providers. There were also two incidents involving infringements by the state security forces whereby they arrested hospital staff and also a wounded member of an armed group undergoing treatment at the RIMS hospital. However, the rest of the incidents were perpetrated by armed groups or “unknown” perpetrators.

This was also seen in subsequent years beyond the years of study. For instance, in July 2010 a medical student of RIMS hospital was abducted while returning from a rural health centre in Saikot which is located in the Churachandpur district.^28^ In September 2010 a lady doctor and a nurse were abducted by unknown gunmen from the Mekola Public Health Centre (PHC) at Bishnupur District by using a fake “emergency call”(Hueyein News Service 2010). Lastly, in December 2010, a government AYUSH (Department of Ayurveda, Yoga Naturopathy, Unani, Siddha and Homoeopathy) doctor was abducted from Leimapokpam Health Centre in Bishnupur District. The perpetrators also took away the mobile phones of the staff of the health centre and also the records of all Tubercolosis (TB) patients.^29^

## Discussion

The physical protection of healthcare workers is of utmost ethical concern. Previous studies that have looked at violence against humanitarian workers have pointed out that, while local workers are bear a greater burden of the violence as compared to expatriates there is significant under-reporting and limited documentation of these cases.^30^^,^ 31 They emphasize the need for creating comprehensive data-bases to study the risk of healthcare personnel in armed conflict situations. The micro-level approach of this study reveals that local health workers are subject to a variety of threats and attacks that violate their personal security and safety. However, they practice their profession in the conflict zone itself outside protected compounds and are witnesses and victims to the instability and violence that is pervasive in these areas. Most importantly, unlike staff members of humanitarian organizations that operate in an institutional framework that invest in security and safety measures; domestic health service providers (such as hospitals and local NGOs) are unable to engage in security risk management as they function under severe resource constraints: both financial and human.

In the case of conflict zones in India (and especially in the area of study-Manipur) the major government-run hospital and staff seem to become a significant target of armed activity. While health service providers seek to remain neutral, it is highly problematic that armed actors view them as legitimate targets. The burden on national health workers is further accentuated as they do not have the option of exiting from the situation despite incidents of direct violence. Thus, the provision of safety and security for health personnel (and institutions) is dependent on individual level strategies as opposed to being organizationally led. This is especially echoed by a study on ambulance drivers in the conflict affected state of Jammu and Kashmir which shows that the drivers place a higher premium on ensuring that patients reach the hospital, than on their own individual safety.^32^ This also leads them to engage in strategies that allow them to navigate security check points as well as cope with the interference and obstructions placed by security forces. However their resilience in the face of danger must not override the fact that they also suffer from high levels of mental stress and insecurity for which there were no corrective measures available.

A recent study by MSF in Syria did highlight the plight of national health workers.^33^ There is however no database or event reporting system, that record attacks specifically on national health workers; and more so in conflict zones where international humanitarian organizations have a sparse presence or are wholly absent. This contrasts with a number of research based studies and databases that draw attention to the seriousness of attacks on international humanitarian aid workers.

What is even more troubling is that the impact of conflict on national health systems is a serious one within the South Asian region itself. However, the lack of research on the subject implies that the scale and intensity of the problem is unknown. The recent targeted killing of six female polio workers and a doctor in Swabi (Pakistan);^34^ and, the killing of a prominent eye surgeon and his son in the Pakistani city of Lahore are reflective of a larger process of the general targeting of health workers in the country.^35^ Similarly during the Maoist insurgency in Nepal the conflict led to artificial barriers being placed on health provision such as blockades and also saw the destruction of health facilities. Another important process experienced in Nepal was the creation of a “rebel” health system that paralleled the government structures.^10^

The paper therefore recommends that a regional level comparative studies on threats to health workers, as well as qualitative studies on resilience of national health workers be conducted. This would allow for the creation of policies or security management practices for national health providers that can be tailored to the local context. Indeed, in the case of India, there is a requirement for the federal and state governments to develop norms for the protection of health providers. However, healthcare in the state must not only been seen as a passive victim to the breakdown of societal structures. In the context of Manipur, the demonstration through evidence, that damages to health institutions and barriers to service provision in the state, have effects that go beyond the “community” one belongs to, can itself be a critical unifying force and can lead to a degree of introspection. Moreover, the security forces, armed groups and ethnic political organizations cannot argue against the legitimacy of the universal right to health care and any political obstruction of heath provision requires mass condemnation. It is critical to shape the discourse on peace-building in the region by going beyond “political” configurations. Thus, by encouraging research that looks at cross-cutting issues that affect all communities and designing solutions for common problems might also hold the key to structural peace. Health services and providers are most greatly and yet most vulnerable in violent conflict, making developing effective ways of protecting them all the more imperative. Health is thus a potential area whereby knowledge driven solutions can be introduced to create a common vision in areas to mitigate the disruptions in social structures and communal amity that are intrinsic to situations of conflict.

## Conclusions

The interaction of conflict dynamics with health systems in the case of Manipur (and the larger North-eastern region) is highly under researched with relevant data and documentation hard to obtain. What is inescapable in the state is the spill over of the “political” dimension into a fundamentally critical and neutral arena as health provision. It is one of our aims that in the broader research on healthcare provision in situations of armed conflict, attention must be drawn to the challenges faced by local health providers. In most situations, local health providers are the forefront of collapsing systems. Moreover, the manner by which health providers continue to function, despite intimidation and attacks, requires further elaboration. Documentation is essential to identify violations, create mechanisms to for protection and develop the political will to enforce them. Further research is essential to understand the other dimensions of this interaction and determine its short-term and long-term impacts.

The aim of this study was to assess longitudinal and spatial trends in incidents involving health care workers in Manipur during the period 2008 to 2009. We have sought to present the constraints on healthcare delivery in a state which is not only experiencing an insurgency, but also inter-ethnic societal disruptions. Our results indicate that insurgency-related incidents in Manipur are increasingly involving health care workers. Although the absolute levels are still quite low, the trend is alarming. Further research is needed to confirm or reject the trend seen here, and if confirmed, should be followed by interventions by central and state government.

## Conflict of Interest

The authors have declared that no competing interests exist.

## Authors' Contributions

SS and SD developed and collated the MMID. SS, SD, MG, and NR designed the study. MG performed the analyses. SS drafted the first version of the manuscript. SD, MG, and NR provided substantial input and edited for important intellectual content. All authors have given their final approval for this version to be published.
